# Supplementary material for: Saffron as a Promising Therapy for Inflammatory Bowel Disease
Source: Nutrients. 2024 Jul 20;16(14):2353. doi: 10.3390/nu16142353 (PMC11280066; doi:10.3390/nu16142353)
Supplement: Supplementary file 1 [file nutrients-16-02353-s001.zip › nutrients-3089132-supplementary.pdf]

Supplementary Table 1: Available therapies for IBD					
**Type of drug**	**Subclass drugs**	**Dosage**	**Conditions**	**Side Effects**	**Cost***
5-aminosalicylic acids (5-ASA)	Mesalazine	oral 1.6-4.8 g per day / 1-4 g suppository	UC/CD	Renal complications, minimal-change nephropathy, Tubulointerstitial nephritis	$2.3/1.2g<br><br>(1.2g (120 tablets) = $274.36)
	Sulphasalazine	1-4 g	UC/CD	Allergic reactions	$00.23/.5 g<br><br>(.5g (120 tablets) = $27.30)
Corticosteroids	Hydrocortisone	enema 100 mg/day,	UC/CD	Hypertension, new onset diabetes mellitus, infection, osteonecrosis, steroid associated osteoporosis, myopathy, psychosis, cataracts, glaucoma, moon face , acne, infection ,ecchymoses , hypertension, hirsutism , petechial bleeding and striae	$06.20/100mg<br><br>(28 enemas (60ml) 100mg/60ml=$174.05)
	Prednisolone	5mg suppository/ 40 mg Oral	UC/CD		$14.60/5mg<br><br>(5mg (30 tablets)= $437.90)
	Budesonide	enema 2mg once daily	UC/CD		$00.01/mg ($865.90/66.8 g)
	Betamethasone dipropionate	5 mg/day enema	UC/CD		$01.20/g<br><br>($18.00/15g)
Antibiotics	Ciprofloxacin 500 mg, orally twice daily and metronidazole 500 mg twice daily, vancomycin 500 mg twice daily .		UC/CD	Intolerance to treatment, Clostridium dificile infection, and increasing antibiotic resistance. ciprofloxacin include tendonitis, tendon rupture, photosensitivity, inhibition of cartilage growth in fetuses and children, oral thrush, and QT prolongation. Metronidazole frequently causes gastrointestinal (GI) disturbances and may cause permanent peripheral neuropathy. Vancomycin is associated with Nephrotoxicity and ototoxicity.	Ciprofloxacin= $01.37/500 mg<br><br>(500mg (14 tablets)= $19.17)<br><br>Metronidazole= $00.80/500 mg<br><br>(500mg (14 tablets)= $11.21)<br><br>Vancomycin= $26.43/500 mg<br><br>(250mg (40 capsules)= $528.60)
Prebiotics	Fermentable carbohydrates(oligosaccharides and inulin)		UC/CD	increase in gastroesophageal reflux ,gaseousness and bloating. Abdominal pain and diarrhea occur with large doses.	
Probiotics	Probiotic preparations include S boulardii, Lactobacillus GG, EcN 1917, and VSL#3.		UC/CD	Diarrhea, abdominal pain, arthralgia, abdominal bloating, and some discomfort.	
Synbiotics	Contain both probiotics and prebiotics		UC/CD		
Thiopurine	Azathioprine	2.5 mg/kg/day	UC/CD	Myelosuppression, nausea, leucopenia, pancreatitis, and risk of lymphoma	$00.85/50 mg

						(50mg (30 tablets)= $25.38)
	Mercaptopurine	1-1.5 mg/kg		UC/CD	Nausea, fever, rash, flu-like symptoms, and arthralgias,Hepatotoxicity,Myelosuppression,Pancreatitis	$01.58/50 mg<br><br>(50 mg (90 tablets)= $141.92)
	Thioguanine	20 mg (range: 20–40 mg)		UC/CD	Nausea, Vomiting, Infection, Bruising, bleeding, Anemia	$260.00/40 mg<br><br>(40mg (25 tablets)= $6496)
Antimetabolites	Methotrexate	25mg SC		UC/CD	Nausea, mucosal ulceration, alopecia, fatigue, fever, increased risk of infection, leukopenia, GI bleeding, pancreatitis, cirrhosis, aplastic anemia, malignancy (lymphoproliferative disorders), infections, interstitial pneumonitis, renal impairment, and teratogenesis.	$01.75/ 25mg<br><br>(2.5 mg (20 tablets) = $35)
Calcineurin inhibitors	Ciclosporin	2mg per kg body weight (total daily dose)		UC	Anaphylaxis, impaired renal function, hirsutism, tremor, hypertension, hepatic dysfunction, fatigue, gingival hypertrophy, gastrointestinal disturbances	$00.77/mg<br><br>(25 mg (30 capsules) = $23)
	Tacrolimus	0.1 mg/kg/dose every 12 hours		UC	Transient renal insufficiency, hyperkalemia, hypertension, Nausea and vomiting, Decreased liver function, Headache, Increased risk of infection, Neurotoxicity, seizures.	$01.49/mg<br><br>(1 mg (100 capsules)= $149)
Advanced medical therapies		induction	maintainance			
	Adalimumab	160 mg day 1, 80 mg day 15 SC	40 mg q2 weeks SC	UC/CD	Injection site reactions , pancytopenia worsening or initiation of congestive heart failure, a lupus-like syndrome, a promotion of lymphoma, medically significant cytopenias, and worsening or initiation of a multiple sclerosis/neurological disease	$697.26/10mg/.2 mL
Biologics-Anti-TNF	Infliximab (IV , SC)	5 mg/kg at 0, 2, 6 wk IV	5 mg/kg q8 weeks IV	UC/CD	Infusion or injection site related anaphylaxis reactions, Upper respiratory tract infections,nausea,headache and diarrhea, hepatotoxicity , neurotoxicity,	$510.00/100mg
	Golimumab	200 mg day 1, 100 mg day 15 SC	100 mg q4 weeks SC	UC	Lupus-like syndrome , hypersensitivity reactions ,Worsening or new-onset demyelinating disorders, worsening CHF symptoms, HBV reactivation, respiratory tract infections	$6435.00/50mg/.5mL
Biologics-anti integrin	Vedolizumab (IV , SC)	300 mg at 0, 2, 6 wk IV	300 mg q8 weeks IV	UC/CD	Nasopharyngitis, oropharyngeal infection, and gastrointestinal infection,Headache,Vomiting,Injection site erythema	$9,135.41/300 mg

anti-IL12/23	Ustekinumab	55 kg: 260 mg ; 55–85 kg: 390 mg ; 85 kg: 520 mg IV	90 mg q8 weeks SC	UC/CD	Respiratory tract infection, Hypersensitivity reactions/anaphylaxis, exacerbation/reactivation of existing infection, Vomiting	$82.35/5 mg
anti-IL23	Risankizumab	600 mg at 0, 4, 8 wk IV	180 mg or 360 mg q8 weeks SC	CD	Hypersensitivity reactions, upper respiratory infection, headache, fatigue, injection site reaction, tinea infection	$20,184/150mg/mL
Janus kinase (JAK) inhibitors	Tofacitinb	10 mg BID for 8 wk PO	5 mg or 10 mg BID; XR dosing 11 mg or 22 mg daily PO	UC	Hypersensitivity reactions, upper respiratory infection, headache, fatigue, Gastroenteritis, Increased blood creatine phosphokinase	$92.80/10mg<br><br>(10mg (30 tablets)=$2784)
	Upadacitinib	45 mg daily for 8 wk (UC), 12 wk (CD) PO	15 mg or 30 mg daily PO	UC/CD	Acne, hypertension, anemia [×2], abdominal pain, and increased liver enzyme levels	$451.81/45 mg<br><br>(45 mg (28 tablets)= $12650.55)
S1P modulators	Ozanimod	0.23 mg daily day 1–4 0.46 mg daily day 5–7 PO	0.92 daily PO	UC	Increased liver function and blood pressure , arrythmias, hypersensitivity	$313.39/.23 mg<br><br>(.23 mg (7 capsules)= $2193.75)
Dietary interventions	Specialist or exclusion diets	Lactose-Free Diet, Gluten-Free Diet, Low FODMAPs Diet, Specific Carbohydrates Diet and Anti-Inflammatory Diet, Mediterranean Diet		UC/CD	Nausea, bloating, or intolerance to the diet, nutrient deficiencies.	
Faecal transplantation	Introducing feces from a well-tested, healthy donor into the gastrointestinal tract of a person with a specific chronic disease			UC/CD	Transient diarrhea, abdominal cramps or pain, low-grade fever, bloating, flatulence, and constipation in long term host susceptibility to diseases, including obesity and immune-mediated disorders, such as immune thrombocytopenia, rheumatoid arthritis, and inflammatory bowel disease	
Surgical therapies	Total proctocolectomy (removal of the rectum and part or all the colon) and ileoanal pouch anastomosis (creation of a J-pouch).Colectomy			UC/CD	Infection, bleeding, and bowel obstruction, needs for lifelong management and other complications, like patient factors (age, overall health, procedure	

	with ileostomy, Colectomy with ileo-rectal anastomosis, Stricturoplasty	specifics, location, and insurance coverage, surgery does not address the underlying immune dysfunction.	

Note: BID, twice daily; CD, Crohn's disease; IBD, inflammatory bowel disease; IV, intravenous; PO, per oral; SQ, subcutaneous; UC, ulcerative colitis; "XR" extended-release formulations; q8 once every 8 weeks; q4 once every 4 weeks; wk ,week ;

**=Used goodrx.com and drugs.com to find average cost of each drug used for IBD-UC/CD*
